# Biorefinery cascade processing for creating added value on tomato industrial by-products from Tunisia

**DOI:** 10.1186/s13068-016-0676-x

**Published:** 2016-12-01

**Authors:** Mouna Kehili, Lisa Marie Schmidt, Wienke Reynolds, Ayachi Zammel, Carsten Zetzl, Irina Smirnova, Noureddine Allouche, Sami Sayadi

**Affiliations:** 1 Laboratory of Environmental Bioprocesses, Centre of Biotechnology of Sfax, University of Sfax, PO Box 1177, 3018 Sfax, Tunisia; 2 Institute of Environmental Technology and Energy Economics, Hamburg University of Technology, Eißendorfer Straße 40, 21073 Hamburg, Germany; 3 Institute of Thermal Separation Processes, Hamburg University of Technology, Eißendorfer Straße 38, 21073 Hamburg, Germany; 4 Ayachi Group Industry, El Mansoura, 6131 Siliana, Tunisia; 5 Laboratory of Chemistry of Natural Substances UR11-ES74, Faculty of Sciences of Sfax, University of Sfax, PO Box 1171, 3000 Sfax, Tunisia

**Keywords:** Biorefinery, Tomato industrial by-products, SC-CO_2_ extraction, Carotenoids, Oil, Protein isolation, Liquid hot water hydrolysis, Lignocellulose

## Abstract

**Background:**

In today’s consumer perception of industrial processes and food production, aspects like food quality, human health, environmental safety, and energy security have become the keywords. Therefore, much effort has been extended toward adding value to biowastes of agri-food industries through biorefinery processing approaches. This study focused, for the first time, on the valorization of tomato by-products of a Tunisian industry for the recovery of value-added compounds using biorefinery cascade processing.

**Results:**

The process integrated supercritical CO_2_ extraction of carotenoids within the oil fractions from tomato seeds (TS) and tomato peels (TP), followed by a batch isolation of protein from the residues. The remaining lignocellulosic matter from both fractions was then submitted to a liquid hot water (LHW) hydrolysis. Supercritical CO_2_ experiments extracted 5.79% oleoresin, 410.53 mg lycopene/kg, and 31.38 mg β-carotene/kg from TP and 26.29% oil, 27.84 mg lycopene/kg, and 5.25 mg β-carotene/kg from TS, on dry weights. Protein extraction yields, nearing 30% of the initial protein contents equal to 13.28% in TP and 39.26% in TS, revealed that TP and TS are a rich source of essential amino acids. LHW treatment run at 120–200 °C, 50 bar for 30 min showed that a temperature of 160 °C was the most convenient for cellulose and hemicellulose hydrolysis from TP and TS, while keeping the degradation products low.

**Conclusions:**

Results indicated that tomato by-products are not only a green source of lycopene-rich oleoresin and tomato seed oil (TSO) and of protein with good nutritional quality but also a source of lignocellulosic matter with potential for bioethanol production. This study would provide an important reference for the concept and the feasibility of the cascade fractionation of valuable compounds from tomato industrial by-products.

**Graphical abstract:**
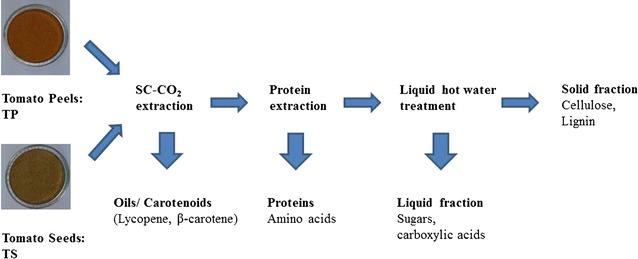
Schema of biorefinery cascade processing of tomato industrial by-products toward isolation of valuable fractions.

## Background

The biorefinery concept is defined as an approach for the generation of value-added products such as biochemicals, biofuels, heat, and electricity from renewable energy sources and particularly from biomass [1–3]. Being available in large quantities and non-competitive with the food industry, the agro-industrial by-products become more and more interesting as raw materials for such refineries [4]. In the context of tomato agriculture, worldwide, million tons are industrially processed yearly resulting in large amounts of residues that are estimated around 4% of the total processed tomatoes [5, 6]. In the last decade, Tunisia was ranked number 9 worldwide for the industrial transformation of tomatoes, with a rate of 36,000 tons of raw tomatoes per day within the summer season. Consequently, high quantities of by-products, ranging between 20,000 and 30,000 tons, were disposed from the Tunisian tomato industry per year, with 56 and 44% being the respective proportions of tomato peels and seeds.

Since these residues contain a large valuable fraction, they could be further treated at the industrial site for the production of bioactive compounds or biofuels, increasing the bioeconomy and solving the problem of pollution connected with tomato processing. Indeed, tomato peels were reported to contain, on dry basis, 14–20% protein [6, 7], 40–74% lignocellulosic material [6–8], and 3–5% oleoresin, being the oil fraction from tomato peels [9, 10]. Besides, tomato seeds were shown to contain 20–40% protein [11, 12], 35–50% lignocellulosic material [7, 13], and 18–37% oil [14, 15]. Nevertheless, currently, valorization of tomato peels has attracted little interest, since they are mostly considered for the extraction of carotenoids, especially lycopene [6], while tomato seeds have attracted very limited attention so far. Effectively, there was a current upsurge concerning carotenoid extraction from tomato peels regarding their significant role in human health by acting as biological antioxidants [16]. In this context, tomatoes were shown to be the major source of dietary lycopene whose concentrations vary from 430 to 2950 mg/kg, on dry basis, with tomato peels containing about five times more lycopene than tomato pulp [17]. Recently, supercritical CO_2_ (SC-CO_2_) has been favored for the extraction of lycopene. Indeed, this green and safe technology is suitable for the recovery of lipophilic substances, e.g., carotenoids, and lipids from numerous plant matrices [18]. Yet, regarding literature, few papers have recently suggested other valorization pathways of tomato by-products such as biofuel production or as a source of oil, polysaccharides, and protein. For instance, Sarkar and Kaul [11] identified a pilot plant setup to extract tomato seeds protein toward its commercial application as a functional additive in food formulations. Additionally, the study by Rossini et al. [19], performed on tomato manufacturing residues, suggested the use of tomato peels for combustion and tomato seeds for mechanical oil extraction. Moreover, based on the fiber analysis of tomato industrial peel by-product, Toscano et al. [8] showed that hemicellulose, cellulose, and lignin dry mass fractions corresponded to 4.8, 22.5, and 46.9%, respectively. This structural composition encouraged Toscano et al. [8] to use this processing residue for the production of a solid biofuel with energy properties similar to those of coal through a torrefaction treatment. Besides, Del Campo et al. [20] revealed that tomato residues could be potential feedstocks for ethanol production not only because of their low cost and high availability but also because of their considerable amount of sugars. In fact, Del Campo et al. [20] found between 40.3 and 50.2% of soluble sugars by weight in the hydrolysate after hydrothermal treatment of tomato residues at temperatures from 100 to 130 °C and reaction times from 5 to 30 min.

Practically, to be effectively used as a raw material in biorefinery for biofuel production, tomato by-products need to be firstly hydrolyzed to fermentable sugars [2, 3, 21]. Recent innovations about lignocellulosic biomass hydrolysis include acid or base treatments, which are not yet satisfactory because of the ecological waste issues [22, 23]. Hydrolysis with hemicellulolytic and cellulolytic enzymes had also been commonly used for lignocellulose degradation [2, 24]. However, enzymatic hydrolysis is time consuming and is not enough to break down the hemicellulose and cellulose complex structures to simple sugars [2, 25, 26]. Interestingly, the hydrolysis with liquid hot water (LHW) , a physicochemical process using pressurized water, is picking up an expanding consideration as an environmentally friendly technique. Effectively, LHW has an extensive variety of applications, such as extraction, hydrolysis, and wet oxidation of organic compounds [4, 25, 27].

Despite the advanced literature, Scoma et al. [28] and Vardanega et al. [29] revealed that reports on multi-target second-generation biorefinery of tomato residues are still very few in literature. Therefore, this paper focuses on using a consecutive biorefinery approach to investigate the potential of tomato industrial by-products of a Tunisian industry for the recovery of value-added products including oleoresin, oil, carotenoids, protein, sugar, and lignin. In a first step, carotenoids were extracted within the lipid fraction, oleoresin from tomato peels and tomato seed oil, using SC-CO_2_. The residues from the SC-CO_2_ extraction step were then submitted to batch protein extraction. LHW hydrolysis was applied for the treatment of solid residues, recovered after SC-CO_2_ and protein extraction for the recovery of hemicellulose sugars. Although the integration of all thermal separation processes in a kind of “one stop unit” is feasible, in this work, it has been decided that the bespoken cascade still contains some transfer interfaces between the different unit operations, for research and analytical purposes. Moreover, for all process steps, the most important process parameters were investigated based on preliminary results. Then, the effect of preliminary protein and oil extraction on LHW hydrolysis was investigated.

With regard to the scale-up feasibility of the processes requiring high pressure, it is noteworthy that the supercritical and compressed fluid extractions have found their origin in the industrial petrorefinery in the 1950s through the Solexol^®^ and the ROSE^®^ (residuum oil supercritical extraction) processes [30, 31]. Recently, these technologies have been also applied in large scale in food and pharmaceutical industries, as the benefits of the process are overweighting the investment costs either in the case of the high-value extracts, e.g., carotenoids and phytopharmaceuticals, or in the case of added consumer value on the residue, e.g., decaffeinated coffee and defatted oil crops for cattle feed. Interestingly, a reproducible trend for cost statements was found in the study by Brunner [32] that reported process costs of approximatively 500–1000 EUR/ton for the treatment of an annual amount of 30,000 tons of solid biomass. In the same way, high-pressure techniques for hot water hydrolysis and steam explosion are nowadays applied in second-generation biorefineries at least in large industrial pilot scale, as can be seen in the processes of Clariant Sunliquid^®^ (Germany), Proesa^®^ (Italy), and Inbicon^®^ (Denmark).

## Methods

### Feedstock

Tomato peels and seeds were recovered from a processing plant of peeled tomato, located in Siliana, Tunisia. The residues from tomato processing were dried under sunlight until a dry matter content of 95 ± 2%. The dried residue, consisting of 35% of tomato seeds and 65% of tomato peels on dry basis, was separated using manual sieves, then ground using a conventional grinder to a maximum particle size of 300 µm, and kept at −20 °C until further use.

### Chemicals

Lycopene, β-carotene, O-phthaldialdehyde (OPA), hexane, and hydrochloric acid were purchased from Sigma Chemical Co. (Sigma-Aldrich Company, St. Louis, MO, USA). Citric acid monohydrate, sodium hydroxide, acetonitrile, dichloromethane, methanol, and sulfuric acid were purchased from Carl Roth GmbH (Karlsruhe, Germany). Ethyl acetate and calcium carbonate were obtained from Merck (Darmstadt, Germany), and disodium phosphate was purchased from Fluka (Buchs, Switzerland). Carbon dioxide (99.5%) purchased from Yara/Praxair GmbH (Neuwied, Germany) was used for the supercritical extraction experiments. Nitrogen (99.95%) from Westfalen AG (Münster, Germany) was used for maintaining pressure in the filling process with liquid hot water.

### Steps of biorefinery cascade processing

#### Supercritical CO_2_ extraction

An analytical and research Spe-ed SFE-2/4 unit (Applied Separations, Allentown, PA, USA) was used. The apparatus was equipped with a stainless steel extraction column (50 cm^3^ of capacity), a back pressure regulator, a cooling unit, a high-pressure pump for CO_2_, and a CO_2_ flow meter. Liquid CO_2_ was passed through a cooling unit and compressed to the operating pressure by a high-pressure pump. Compressed CO_2_ was fed continuously into the extractor maintained at the operating pressure and temperature. 10 g of ground tomato biomass (peels or seeds) was loaded into the extraction vessel. The SC-CO_2_ extraction was run under 80 °C, 400 bar, and 4 g CO_2_/min for 2 h. At the outlet of the extractor, the fluid was expanded to the atmospheric pressure and the carried solute was collected, each 30 min, in a preweighed glass vial. The different glass vials were weighed to determine the amount of oleoresin and oil extracted from tomato peels and seeds. The vials were then kept at −20 °C for further analysis of the carotenoid contents (lycopene and β-carotene) using high-performance liquid chromatography (HPLC). The supercritical fluid extraction (SFE) residues of peels and seeds were weighed and considered for the next step of protein batch extraction. This step was repeated twice under the same set of conditions.

#### Protein extraction

In this step, batch protein extraction was performed according to the protocol applied by Gairola [33], who extracted proteins from brewers’ spent grains, with some modifications. A quantity of 10 g of SFE residues was added to 200 mL of NaOH (0.05 M) and maintained at 90 °C for 30 min under magnetic stirring at 700 rpm. Once cooled down, this solution was centrifuged at 4500 rpm, for 1 h at 25 °C. The raffinate phase, separated solid, was subjected to the next step of LHW hydrolysis, and the supernatant was considered for protein precipitation by decreasing pH to a value of 4 ± 0.1 with citric acid (0.5 M) solution. The mixture was centrifuged again at 4500 rpm, for 1 h at 25 °C, and the raffinate was considered as the final protein extract. For comparison, protein extraction was similarly carried out starting from raw samples of tomato peels and seeds, not submitted to a previous SFE step. Protein extraction was performed twice starting from raw and pretreated residues.

#### Liquid Hot Water hydrolysis

LHW hydrolysis was carried out in stainless steel reactors with a volume of 45 mL each (High Pressure Reactor BR-25, Berghof, Eningen, Germany). The temperature inside the reaction mixture was controlled over the heating jackets as described also by Gairola and Smirnova [34]. In each reactor vessel, 0.6 g of tomato biomass (dry matter) was adjusted to 30 g with distilled water inside a polytetrafluoroethylene (PTFE) cartridge. Nitrogen was used to pressurize the reactor to 50 bar. The reaction was carried out for 30 min at three different temperatures (120, 160, and 200 °C). The reaction was stopped using an iced water bath. Kazan et al. [2] and Mohan et al. [25] suggested similar LHW operating time for optimum olive pomace and bamboo hydrolysis. Afterwards, the hydrolysate and the solid residue were separated by centrifugation at 4500 rpm and 25 °C for 1 h. The solid fraction was dried at 60 °C prior to the compositional analysis of their lignin, cellulose, and hemicellulose contents [26]. The supernatant was collected and analyzed for its composition in organic acids, sugars monomers, and oligomers. For comparison, LHW hydrolysis was similarly carried out starting from raw samples of tomato peels and seeds which were not submitted to a previous SFE and protein extraction steps. Each experiment was carried out in duplicate.

### Analytical methods

#### Initial lipid contents in tomato by-products

5 g of dry biomass and 200 mL of hexane were used for the extraction of the maximum oleoresin and oil contents in tomato peels and seeds using Soxhlet extraction for 12 h [35]. During extraction, the Soxhlet extractor was covered with an aluminum foil to prevent oxidation and degradation of the valuable carotenoids. The Soxhlet extraction was carried out in triplicate and the extracts were dried using a rotary evaporator. The lipid fraction mass, extracted from both samples, was determined gravimetrically and considered as 100% extraction yields of oleoresin and oil. These total lipid fractions, oleoresin from tomato peels and tomato seed oil, were dissolved in ethyl acetate, filtrated using 0.22-μm hydrophobic PTFE membrane filters, and analyzed for their total lycopene and β-carotene contents using HPLC as detailed below [35].

#### Lycopene and β-carotene quantification

The obtained extracts from tomato peels and seeds, oleoresin and tomato seed oil, respectively, were analyzed using HPLC for their lycopene and β-carotene contents. SFE extracts, dissolved in ethyl acetate, were analyzed using a Ginkotek HPLC system equipped with an Agilent eclipse XDB-C18 column (5 μm; 4.6 mm × 150 mm) and a UV–Vis detector (SPD-GAV). An isocratic mode was applied for the elution of carotenoids using acetonitrile/dichloromethane mixture (75:25; v/v). Chromatographic separation of the samples was performed at a constant flow rate of 1.5 mL/min and the absorption wavelength was set at 470 nm. In the extracts, lycopene and β-carotene were identified and quantified by comparing their retention times and peak areas with their respective standards analyzed under the same conditions [17]. All samples were analyzed in duplicate.

#### Protein analysis

The protein content was calculated based on the nitrogen content estimated with Kjeldahl method, for the solid fractions, multiplied by the specific factors equal to 6.67 for tomato peels and 6.46 for tomato seeds. Herein, the specific factors were determined based on the amino acid analysis of the protein extracts from both tomato residues. Since we aimed to determine the mass balance of protein within the different fractions after the protein extraction step, the protein content in the aqueous fraction was determined using the Analytic Jena multi N/C 3100 instrument (Jena, Germany). Besides, the composition of the protein extracts in amino acids was assessed after hydrolysis with 6 M HCL at 115 °C for 16 h, derivatization with O-phthaldialdehyde (OPA), and analysis with reversed-phase HPLC. The HPLC was equipped with a ZORBAX Eclipse C18 column (3.5 μm; 4.6 mm × 150 mm) and a fluorescence detector (Jasco FP1520) connected to a Chromeleon data system (Thermo Scientific). The temperature of the HPLC column was maintained at 21 °C. Gradient elution was applied using Na_2_HPO_4_ buffer (0.04 M), pH 7.8, and a mixture of acetonitrile/methanol/water, 45/45/10 (v/v), at a flow rate of 2 mL/min.

The nutritional quality of the protein from tomato residues was estimated based on two parameters: amino acid scoring pattern and protein efficiency ratio (PER) according to Sarkar and Kaul [11]. The amino acid score was calculated by dividing the content of each amino acid by a reference amino acid pattern of a protein recognized for the nutrition of preschool children (1–2 years old) [36]. Moreover, the PER was calculated as an average of the following three equations with amino acids contents measured as g/100 g protein [11]:

PER = −0.684 + 0.456 × (Leu) − 0.047 × (Pro)

PER = −0.468 + 0.454 × (Leu) − 0.105 × (Tyr)

PER = −1.816 + 0.435 × (Met) + 0.78 × (Leu) + 0.211 × (His) − 0.944 × (Tyr).

#### Liquid fraction analysis after liquid hot water treatment

After LHW treatment, the liquid fractions were analyzed for their sugar monomer and oligomer contents as well as sugar degradation products such as furfural, hydroxymethylfurfural (HMF), and organic acids using a HPLC system (1200 Agilent Technologies) with refraction index detection (1100 Agilent Technologies). The HPLC analysis was performed at 40 °C using dilute H_2_SO_4_ (0.025%) as an eluent at a flow rate of 0.3 mL/min. Analytical hydrolysis was carried out for 60 min at 121 °C with 4% H_2_SO_4_. The samples were neutralized with CaCO_3_, centrifuged, and analyzed again for their sugar content using HPLC. For calculation, xylose, arabinose, galactose, mannose, rhamnose, and lactose were assigned to the hemicellulose fraction, whereas the complete amount of glucose, fructose, and cellobiose was considered as cellulose [25, 37]. Additionally, as part of the hexoses and pentoses are converted to other by-products, these sugar transformation by-products were calculated as the percentage of the initial amount of hemicellulose and cellulose contained in the solid sample submitted to the LHW experiment. The sugar transformation by-products include HMF, furfural, levulinic acid, formic acid, and lactic acid.

#### Solid fraction analysis after liquid hot water treatment

For acid-insoluble lignin determination and sugar analysis, the solid samples were treated using a two-step acid hydrolysis [37, 38]. The biomass was mixed with 72% H_2_SO_4_ (w/w) and hydrolyzed for 1 h. Subsequently, water was added for diluting the H_2_SO_4_ solution to 4% and the sample was boiled for 40 min [2]. The solid residue was filtered, dried, and weighed to determine the acid-insoluble lignin content. The sugars were quantified within the hydrolysates by HPLC.

## Results and discussion

### Supercritical CO_2_ extraction

**Fig. 1:**
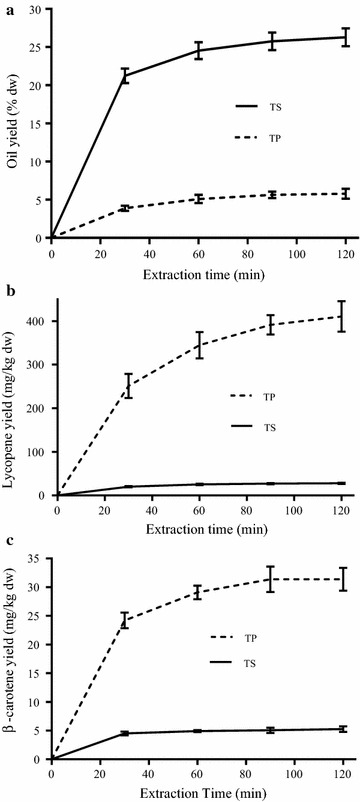
Variation of oil (**a**), lycopene (**b**), and β-carotene (**c**) extraction yields from dry weights (dw) of tomato peel (TP) and tomato seed (TS) by-products using SC-CO_2_ at 400 bar, 80 °C, and 4 g CO_2_/min for 120 min using a batch of 10 g of biomass with 300 μm particle size

The first step in the biorefinery cascade approach aimed to extract the carotenoids within oleoresin and oil from ground tomato peels (TP) and seeds (TS), respectively, using supercritical CO_2_. Figure 1a shows the profile of the extraction yields of oleoresin from TP and oil from TS expressed as the percentage of the respective cumulative extracted mass to the initial dry mass, over time. After 120 min of SC-CO_2_ extraction, about 95.07 and 98.61% of the initial oleoresin and oil contents in TP and TS were recovered, respectively, relating to the extraction of 5.79 ± 1.33% oleoresin from dry TP and 26.29 ± 3.30% oil from dry TS.

In comparison with literature, the present result exhibits higher oleoresin extraction yield (95.07%) than that of 31.78% found by López-Cervantes et al. [10] using ultrasound-assisted extraction of oleoresin from over-ripe tomato using acetone, with the original oleoresin content being equal to 4.50 ± 0.10%. Besides, the yield of tomato seed oil (TSO) extracted in the present study was found to be much higher compared to that found in the study of Eller et al. [39]. Actually, the latter research extracted 17.3% of TSO representing 75% of the maximum extractable oil from dry TS using SC-CO_2_ at 80 °C, 552 bar, and a flow rate of 300 g/min within an extraction cell holding 1.5 kg of ground TS. The difference between results can be attributed to the solvent-to-TS ratio that is equal to 0.4 g CO_2_/g.min in the present study, while that reported by Eller et al. [39] was equal to 0.2 g CO_2_/g.min. In fact, an increase in CO_2_ flow rate seemed to increase the mass transfer of the mediated solutes in CO_2_, enhancing their recovery yields [16].

Figure 1b summarizes the cumulative lycopene extraction yields from TP and TS using SC-CO_2_. Actually, at the end of the extraction time, 410.53 ± 35.12 mg lycopene/kg TP was extracted. This yield represents 60% of the initial lycopene amount equal to 684.23 ± 19.21 mg/kg TP. The present result is comparable with that reported by Machmudah et al. [17] who reached a lycopene extraction yield of 56% of the initial lycopene amount, corresponding to 459.20 mg/kg of dry tomato by-product input. The latter study [17] used SC-CO_2_ extraction in the presence of tomato peels/seeds ratio of 37/63, run under 400 bar, 90 °C, and a CO_2_ flow rate of 4 mg/min for 180 min using 4 g of material. Besides, about 82.22% of the initial lycopene content in TS was recovered after 120 min of SC-CO_2_ extraction, relating to the extraction of 27.84 ± 1.33 mg lycopene/kg dry TS (Fig. 1b). Hence, the TSO recovered at the output of the SC-CO_2_ extraction was rich in lycopene whose concentration was equal to 105.89 ± 13.43 mg/kg TSO.

Furthermore, about 58.82 and 90% of the initial β-carotene contents in TP and TS were recovered after 120 min of SC-CO_2_ extraction, relating to the extraction of 31.38 ± 1.98 and 5.25 ± 0.48 mg β-carotene/kg of dry TP and TS, respectively (Fig. 1c). As a result, the β-carotene content in TSO was about 19.97 ± 1.80 mg/kg TSO which is comparable with the value reported by Müller et al. [12]. Indeed, among the few studies interested in the content of carotenoids in TS and TSO, Müller et al. [12] found that TSO contained 95.60 ± 3.60 mg lycopene/kg and 16.60 ± 0.40 mg β-carotene/kg TSO.

Obviously, the final extraction yields of lycopene and β-carotene are much higher starting from TS as compared to those from TP, more likely because of the high amount of oil in TS. Effectively, the oil content may have induced a co-solvent effect enhancing the solubility and the recovery yields of carotenoids that are highly lipophilic molecules [17]. Therewith, the major amount of the extracted oil and carotenoids from TP and TS, exceeding 60–85% of the final extracted amounts, were recovered during the first 30 min of the extraction time, in all cases (Fig. 1). Indeed, the short extraction time is one of the main promoting aspects of SC-CO_2_ extraction reducing the processing costs while producing a safe and solvent-free extract [40].

In the same context, the study by Prado et al. [41] compared low-pressure solvent extraction (LPSE) with SC-CO_2_ extraction of carotenoids from a large number of vegetable sources. The LPSE included the Soxhlet, agitation, homogenization, shaking, ultrasound-assisted extraction, and centrifugal extraction. In their review [41], they obviously revealed that a large margin of processing times can be considered for each extraction method depending on the raw material selected and all other operating parameters applied. Hence, none of the reviewed approaches can be rated as the best answer for every extractable compound and raw material. Nevertheless, a large number of studies suggested a positive trend toward using SC-CO_2_ extraction for the recovery of carotenoids mainly regarding the growing restrictions imposed on the use of most organic solvents for the extraction of food products [41, 42]. Besides, the advantages of SC-CO_2_ extraction over the conventional methods include the high diffusivity of compressed CO_2_ as well as the possibility and the ease of monitoring the fluid dynamics and the extraction kinetics through the variation of solvent velocity, for instance. Thus, the process costs and equipment characteristics can be set based on the cumulative extraction plots of a specific compound from a given material input, as highlighted by Del Valle et al. [43].

### Protein extraction

Raw TP and TS as well as SC-CO_2_-treated TP and TS residues were considered for protein quantification, extraction, and analysis. The initial protein content was equal to 13.28 ± 3.21% in raw TP and 39.26 ± 1.15% in raw TS, on dry basis. Comparably, Persia et al. [13] and Sarkar and Kaul [11] reported that the protein content in TS ranged between 24 and 40% on dry basis depending on the environmental and genetic varieties. Considering the pretreated tomato by-products with SC-CO_2_, protein contents showed negligible difference with that of raw samples for both TP and TS. The protein extraction yields from pretreated TP and TS, expressed as the percentage of their respective initial protein contents, were 27.37 ± 0.98% and 35.82 ± 2.22%. Starting from raw TP and TS, the protein extraction yields were comparable with those of pretreated samples. However, the protein concentration within the final protein extracts was much higher starting from pretreated TP and TS as compared to that from raw TP and TS. In fact, the protein fraction was 35.83 ± 1.95% from pretreated TP and 80.90 ± 2.56% from pretreated TS, while it was 27.41 ± 2.13% from raw TP and 55.25 ± 3.24% from TS, on dry protein extract basis. Hence, the defatting step using SC-CO_2_ seemed to be useful for better purity of the protein isolates mainly for TS, probably because the fact of removing the lipid fraction from tomato by-products may have led to fewer impurities in the aqueous protein extracts. Likewise, Sarkar and Kaul [11] performed protein isolation from defatted tomato ground seeds (seed meal) by salt extraction. In this study [11], they revealed a higher yield of protein extract that was equal to 27.3% of dry tomato seed meal, representing 68.45% of the initial protein content, with a crude protein concentration of 91.66% in the protein isolate.

It is worth noting, based on the mass balance of protein, that around 15% of the initial protein amount was found within the supernatant after protein precipitation and that the major protein fraction, exceeding 50% of the initial protein amount, was recovered in the solid fraction after the first centrifuge of protein solution. This fact can be considered for further optimization of the protein recovery yield within the final extract using, for instance, a two-step protein extraction as suggested in the study by Zhang et al. [44]. Indeed, in this study [44], they provide a good reference for the optimization of critical parameters toward a cost-effective alkaline extraction of a high protein yield equal to 85% of the initial protein content in green tea residue, with a purity of 52%.

The total amino acid profiles of the protein isolates from TP and TS are summarized in Table 1. The protein fraction extracted from TP was highly rich in glutamic acid (16.38 ± 0.55% of total amino acid content), aspartic acid (11.41 ± 0.52%), serine (9.60 ± 0.34%), phenylalanine (8.70 ± 0.29%), and leucine (7.01 ± 0.37%). TP protein contained also alanine, tryptophan, and isoleucine with values around 5% of the total protein content.

**Table 1:** Total amino acid profile of the protein extracted from tomato peel and seed by-products

	Amino acid (mg/g protein)	
	TP	TS
Aspartic acid	114.1 ± 5.2	102.1 ± 3.7
Glutamic acid	163.8 ± 5.5	214.2 ± 0.7
Asparagine	16.9 ± 1.2	0.0
Serine	96.0 ± 3.4	54.7 ± 2.2
Glutamine	0.0	2.6 ± 0.3
Histidine^a^	20.3 ± 1.8	26.1 ± 0.8
Glycine	45.2 ± 0.8	55.2 ± 3.4
Threonine^a^	35.0 ± 4.2	33.9 ± 2.6
Arginine	44.1 ± 3.4	91.7 ± 4.4
Alanine	56.5 ± 3.1	47.9 ± 3.0
Tyrosine	27.1 ± 0.4	41.2 ± 2.0
Valine^a^	42.9 ± 0.5	52.1 ± 3.9
Methionine^a^	13.6 ± 0.9	18.8 ± 4.1
Tryptophan^a^	57.6 ± 1.7	17.2 ± 0.6
Phenylalanine^a^	87.0 ± 2.9	70.3 ± 4.9
Isoleucine^a^	56.5 ± 1.3	56.8 ± 3.4
Ornithine	21.5 ± 1.5	2.6 ± 0.5
Leucine^a^	70.1 ± 3.7	80.3 ± 5.6
Lysine^a^	31.6 ± 2.5	32.3 ± 3.1

^a^Refers to essential amino acids

Similarly, TS protein was primarily rich in glutamic acid (21.42 ± 0.07%), aspartic acid (10.21 ± 0.37%), arginine (9.17 ± 0.44%), leucine (8.03 ± 0.56%), and phenylalanine (7.03 ± 0.49%). TS were also reported by Persia et al. [13] to be mostly rich in glutamic acid and aspartic acid. Quite comparable but lower amounts of serine, glycine, valine, and isoleucine were nearing 5% of the total TS protein. Unlike many other plant proteins, TP and TS are not deficient in lysine since they contained, respectively, 3.16 ± 0.25 and 3.23 ± 0.31% lysine of the total amino acids. Overall, the essential amino acids, followed by superscript stars in Table 1, represented high proportions of the total amino acids, being equal to 41.46 ± 0.19 and 38.78 ± 0.29%, in TP and TS, respectively.

Besides, TP and TS were deemed to be endowed with good-quality proteins regarding the high scores for most of their essential amino acids as compared to those of a reference protein recognized for the nutrition of infants between 1 and 2 years old [36] (Table 2). Noticeably, tomato by-products are considerably rich in isoleucine, tryptophan, and aromatic amino acids (phenylalanine and tyrosine). Additionally, the protein efficiency ratio, PER, of TP was equal to 2.35 and that of TS was equal to 2.55. As any protein having PER higher than 2.50 is considered to be of high nutritional quality [11], the TS protein can be considered as a good-nutritional quality protein. The TP protein was endowed with a slightly lower quality than that of TS according to the PER rapid estimation. Sarkar and Kaul [11] found a comparable PER equal to 2.66 for TS protein.

**Table 2:** Amino acid scoring pattern of the protein extracted from tomato peel and seed by-products

Amino acids	Reference protein (mg/g protein) [36]	TP protein (mg/g protein)	TP amino acid score (%)	TS protein (mg/g protein)	TS amino acid score (%)
Histidine	18.0 ± 1.4	20.3 ± 1.8	112.8 ± 10.0	26.1 ± 0.8	145.0 ± 4.4
Isoleucine	31.0 ± 0.7	56.5 ± 1.3	182.3 ± 4.2	56.8 ± 3.4	183.2 ± 11.0
Leucine	63.0 ± 1.4	70.1 ± 3.7	111.3 ± 5.9	80.3 ± 5.6	127.5 ± 8.9
Lysine	52.0 ± 2.8	31.6 ± 2.5	60.8 ± 4.8	32.3 ± 3.1	62.1 ± 6.0
Methionine + cysteine	25.0 ± 0.7	13.6 ± 0.9	54.4 ± 3.6	18.8 ± 4.1	75.2 ± 16.4
Phenylalanine + tyrosine	46.0 ± 3.5	114.1 ± 3.3	248.0 ± 7.2	111.5 ± 6.9	242.4 ± 15.0
Threonine	27.0 ± 1.4	35.0 ± 4.2	129.6 ± 15.6	33.9 ± 2.6	125.6 ± 9.6
Tryptophan	7.0 ± 0.3	57.6 ± 1.7	822.9 ± 24.3	17.2 ± 0.6	245.7 ± 8.6
Valine	41.0 ± 0.7	42.9 ± 0.5	104.6 ± 1.2	52.1 ± 3.9	127.1 ± 9.6
Essential amino acids		414.6 ± 19.5		387.8 ± 29.0	

### Liquid hot water hydrolysis

The final step in the biorefinery cascade processing performed in the present study consisted of the LHW treatment of TP and TS residues resulting from SC-CO_2_ and protein extractions. For comparison, raw TP and TS were analyzed under the same operating conditions. According to fiber analysis, the lignocellulosic matter was the major fraction of TP and TS with the values equal to 67.40 ± 2.15 and 63.30 ± 1.06% on dry basis, respectively. In fact, TP contained 18.50 ± 0.13% of dry mass as cellulose, 6.0 ± 0.04% hemicellulose, and 42.90 ± 1.98% as total lignin, being the sum of acid-soluble lignin (2.90 ± 0.13%) and acid-insoluble lignin (40.0 ± 2.01%). TS contained 9.0 ± 0.87% of dry mass as cellulose, 16.20 ± 0.97% hemicellulose with mannose being the major sugar accounting for 11.20 ± 0.66% of TS dry mass, and 38.1 ± 0.78% as total lignin, where the acid-insoluble lignin was 31.0 ± 0.86%. Comparably, Navarro-González et al. [45] showed that the lignocellulosic matter was equal to 84.16% of TP fiber where the major fraction, the insoluble dietary fiber (71.82%), was mainly formed by cellulose and hemicellulose. Besides, the neutral sugar composition of the lignocellulosic matter showed that the main sugars of TP fiber are mannose, galactose, xylose, and arabinose, which indicated that hemicelluloses were the predominant polysaccharides of this fiber. However, this study suggested limited content of lignin in TP fiber [45]. Likewise, Cepeda and Collado [46] showed that the lignocellulosic matter represented 65% of the total tomato fiber, where the soluble dietary fiber was 9% including a pectin content of 6% and the insoluble dietary fiber was 56% including a lignin content of 23%.

### Liquid hot water hydrolysis of tomato peels

LHW hydrolysis experiment consisted of submitting raw tomato peels (TP) and tomato peels previously submitted to carotenoid extraction using SC-CO_2_ and protein extraction (PT-TP) to a batch LHW hydrolysis experiment. Pressurized hot water at 50 bar was used for the pretreatment of TP samples at 120, 160, and 200 °C for 30 min. The slurry was then centrifuged and separated into two parts: solid and liquid fractions that were analyzed for their fiber and sugar composition profiles. Yields mentioned in Table 3 are calculated as the percentages of the initial cellulose, hemicellulose, and lignin amounts contained in the starting material used for the LHW experiment.

**Table 3:** Lignocellulosic component recovery in the liquid and solid fractions of LHW hydrolysates from tomato peels

Sample	Component recovery in LHW liquid fraction (% of total component amount)			Component recovery in LHW solid fractions (% of total component amount)				Weight loss (DW %)
	Cellulose	Hemicellulose	Sugar transformation by-products	Cellulose	Hemicellulose	Acid-soluble lignin	Total lignin	
TP-120	34.9 ± 4.2	0	16.5 ± 3.8	81.8 ± 1.4	80.8 ± 2.7	71.4 ± 3.6	94.6 ± 2.6	21.9 ± 1.8
TP-160	32.9 ± 3.0	7.6 ± 1.5	17.2 ± 2.0	83.9 ± 3.4	68.5 ± 3.0	69.6 ± 2.7	100 ± 5.4	24.9 ± 2.4
TP-200	3.4 ± 0.4	11.5 ± 2.4	22.5 ± 1.8	79.4 ± 3.3	42.3 ± 4.5	69.2 ± 0.8	100 ± 3.9	31.5 ± 0.9
PT-TP-120	2.8 ± 0.7	1.9 ± 0.6	3.4 ± 0.4	75.2 ± 1.0	73.5 ± 5.2	36.0 ± 3.6	87.9 ± 2.0	9.1 ± 4.1
PT-TP-160	2.1 ± 0.1	8.9 ± 1.5	5.8 ± 0.6	62.3 ± 1.6	56.9 ± 3.1	19.4 ± 1.8	75.6 ± 1.7	25.8 ± 1.3
PT-TP-200	0.1 ± 0.04	2.4 ± 0.6	3.1 ± 0.2	67.3 ± 3.1	46.1 ± 2.4	20.7 ± 1.1	80.9 ± 0.7	22.9 ± 0.6

*DW* dry weight

Regarding the liquid fraction analysis shown in Table 3, it is noticeable that although being endowed with a more rigid structure than hemicellulose, cellulose was much more hydrolyzed from raw TP at 120 and 160 °C. Actually, it is undeniable that assuming all the amount of glucose to account for cellulose remains a relative assumption since hemicellulose may always contain glucose [45]. Generally, the release of sugars from cellulose had been reported to begin at 200 °C, whereas in the present study it seemed to occur at lower temperature. Likewise, the study by Mohan et al. [25] revealed that cellulose had undergone hydrolysis at 170 °C. Comparable results were obtained by Lu and Saka [47] during their study on the hydrolysis of Japanese beech in a batch reactor at 170 °C, whereas in a semi-batch reactor the hydrolysis temperature was around 210 °C. Hence, from the study by Lu and Saka [47], it was deducible that a batch reactor configuration allows the use of low operating temperatures, giving a safe and economically favorable operation.

In addition, based on sugar analysis in the solid fractions, the degradation of cellulose seemed quite independent of temperature, while the degradation of hemicellulose increased with increasing temperature. Indeed, quite comparable yields of cellulose, 81.8 ± 1.4, 83.9 ± 3.4, and 79.41 ± 3.3%, were recovered in the solid fractions after LHW hydrolysis of raw TP at 120, 160, and 200 °C, respectively. Whereas 80.8 ± 2.7% of hemicellulose was recovered in the solid fraction after LHW treatment of raw TP at 120 °C, only 68.5 ± 3.0 and 42.3 ± 4.5% of hemicellulose were recovered after LHW treatment at 160 and 200 °C, respectively. Comparable trends were noticed for the solubilization of cellulose and hemicellulose starting from PT-TP, mainly based on the analysis of the solid fractions, where the effect of temperature was more significant for the degradation of hemicellulose than for cellulose (Table 3).

After LHW hydrolysis of raw TP and PT-TP, the highest recovery yields of hemicellulose and cellulose sugars within the liquid fractions were achieved at a temperature not exceeding 160 °C. Overall, further increase of temperature to 200 °C led to a sharp decrease of hemicellulose and mainly cellulose recovery yields within the liquid fractions in favor for the formation of higher sugar degradation by-products from raw TP and PT-TP [2, 48]. Hence, a temperature of 160 °C was deduced to be the most convenient for better cellulose and hemicellulose hydrolysis with LHW treatment of raw TP and PT-TP while keeping the sugar transformation by-products low. Although the fraction of acid-soluble lignin was considerably dissolved during LHW hydrolysis of raw TP, 30.8 ± 0.8% at 200 °C, and PT-TP, 79.3 ± 1.1% at 200 °C, the acid-insoluble lignin was barely hydrolyzed. Indeed, about 75–100% of total lignin was recovered within the solid fractions after LHW hydrolysis of raw TP and PT-TP at different temperature levels. In the same vein, it is worth noting that the medium tended to be more acidic with increasing LHW temperature. Practically, increasing temperature promotes the liberation of acids from hemicellulose such as glucuronic acid and acetic acid, the sugar fermentation products such as lactic acid, and the sugar transformation products such as levulinic acid and formic acid [3]. Therefore, the acid-soluble lignin dissolved more sharply with the increase of the LHW temperature as compared to the acid-insoluble lignin.

### Liquid hot water hydrolysis of tomato seeds

LHW was applied on raw TS and pretreated TS (PT-TS) at the temperatures of 120, 160, and 200 °C and a pressure of 50 bar for 30 min of hydrolysis time. Based on sugar analysis of the liquid fractions from raw TS and PT-TS, it seemed that cellulose was much more hydrolyzed than hemicellulose at different temperatures (Table 4). As for TP, regarding the solid fractions of raw TS and PT-TS, the degradation of cellulose seemed to vary barely as a function of temperature, while hemicellulose degradation seemed to increase significantly with increasing temperature. This can be attributed to the fact that the hydrolysis capacity of water, in LHW treatment, increased with increasing temperature in the subcritical range (P < 221 bar, T < 374 °C) [25]. Effectively, Cepeda and Collado [46] showed that the sterilization of a suspension of tomato fibers in water (5%, w/v) at 110 °C for 20 min led to a reduction in the insoluble dietary fiber content. This reduction could be attributed to partial degradation of cellulose, hemicellulose, and Klason lignin into simple carbohydrates as a consequence of heat treatment. Likewise, the transformation of sugars into other by-products seemed to increase with temperature (Table 4).

**Table 4:** Lignocellulosic component recovery in the liquid and solid fractions of LHW hydrolysates from tomato seeds

Sample	Component recovery in LHW liquid fraction (% of total component amount)			Component recovery in LHW solid fractions (% of total component amount)				Weight loss (DW %)
	Cellulose	Hemicellulose	Sugar transformation by-products	Cellulose	Hemicellulose	Acid-soluble lignin	Total lignin	
TS-120	42.0 ± 4.4	0	5.7 ± 0.5	69.8 ± 1.2	90.2 ± 3.7	90.7 ± 1.8	81.9 ± 3.2	20.9 ± 1.9
TS-160	51.3 ± 2.3	1.5 ± 0.2	6.9 ± 0.7	75.9 ± 2.6	81.6 ± 2.8	68.2 ± 5.2	80.3 ± 1.7	32.1 ± 2.5
TS-200	15.5 ± 3.8	0	11.9 ± 0.8	65.4 ± 3.4	28.4 ± 3.3	26.7 ± 3.6	67.6 ± 2.2	56.9 ± 0.8
PT-TS-120	0	0.5 ± 0.04	2.4 ± 0.3	59.5 ± 2.1	81.5 ± 0.9	33.9 ± 0.9	31.9 ± 4.1	12.5 ± 0.7
PT-TS-160	4.9 ± 0.7	0.5 ± 0.03	2.3 ± 0.2	58.8 ± 3.5	69.2 ± 2.1	24.7 ± 2.9	28.7 ± 2.6	24.4 ± 1.2
PT-TS-200	11.3 ± 1.6	0	5.7 ± 0.5	58.2 ± 4.3	59.6 ± 3.0	8.8 ± 3.4	32.4 ± 2.0	34.6 ± 1.6

*DW* dry weight

LHW hydrolysis results starting from raw TS were compared with those from PT-TS (Table 4). Less recovery yields of cellulose and hemicellulose were obtained in the PT-TS solid fractions as compared to those of raw TS. This is expected to reflect a better hydrolysis of the PT-TS samples and a better sugar recovery within the liquid hydrolysates. However, regarding the results of the relevant liquid fractions of PT-TS, there were low yields of cellulose and hemicellulose sugars. The overall balance of cellulose and hemicellulose recovered within the liquid and the solid fractions from the PT-TS is much less than 100%. Then, it is noteworthy that, besides sugar degradation occurring during LHW hydrolysis, part of the sugars might have already been dissolved in the previous steps of pretreatment, more probably within the protein extraction step. In this context, Jiang et al. [48] suggested that part of the sugars might undergo a transformation into gaseous compounds with increasing temperature, resulting in the decreased amounts of hydrolyzed sugars within the liquid hydrolysates. This may have occurred in the present study, which may explain part of the loss of mass balance of cellulose and hemicellulose.

Furthermore, although lignin is well known for its very rigid structure, lignin polymers have undergone a degradation reaction as a function of temperature. Indeed, increasing the temperature from 120 °C up to 200 °C led to the decreasing recovery yields of acid-soluble lignin from 90.7 ± 1.8 to 26.7 ± 3.6% and of total lignin from 81.9 ± 3.2 to 67.6 ± 2.2% within the solid fractions of raw TS, respectively. Comparably, lignin has also been observed by Jiang et al. [48] and Reddy et al. [27] to undergo solubilization reactions during LHW pretreatment of giant reed and sugarcane bagasse, respectively.

Overall, the total solubilization of TS samples using LHW process increased significantly with increasing temperature from 20.9 ± 1.9% at 120 °C up to 56.9 ± 0.8% at 200 °C, on dry TS basis (Table 4). Similarly, starting from PT-TS, the total solubilization of dry pretreated samples was 12.5 ± 0.7% at 120 °C and 34.6 ± 1.6% at 200 °C. The decrease of the solubilization yields of PT-TS after LHW as compared to that from raw TS can be attributed to the fact that an important fraction of the dry matter had already been removed or partially degraded within the SC-CO_2_ and the protein extraction steps. Indeed, under the effect of heat and pressure during the LHW treatment of raw TS, protein is highly expected to dissolve within the liquid hydrolysates. Besides, despite the fact that oil is not highly extractable with water, some oil droplets were practically noticed within the liquid fractions of LHW slurries from raw TS.

To sum up, the highest recovery yields of hemicellulose and cellulose within the liquid fraction of raw TS after LHW were obtained at 160 °C with the values equal to 1.5 ± 0.2 and 51.3 ± 2.3%, respectively. Although a temperature of 200 °C was found to yield better cellulose hydrolysis from the PT-TS with a value of 11.3 ± 1.6%, a low operating temperature of 160 °C was similarly required for better hemicellulose hydrolysis with a value of 0.5 ± 0.03%. Further increase in the temperature to 200 °C tended to promote the degradation of the hydrolyzed sugars and their transformation to side by-products during the hydrolysis process (Table 4). Indeed, considering the LHW hydrolysis of raw TS and PT-TS, a temperature of 200 °C seemed to promote the transformation of the total hydrolyzed hemicellulose sugars to other by-products, with null hemicellulose sugars recovered within the liquid fractions, in both cases. Indeed, the study by Jiang et al. [48] revealed also that the increased amount of degradation products was consistent with the decrease of hemicellulose recovery efficiency. Thus, 160 °C seemed to be the most convenient temperature for better cellulose and hemicellulose hydrolysis with LHW treatment of raw TS and PT-TS while keeping the sugar transformation by-products low. Quite comparable temperatures equal to 190 and 180 °C were deduced by Lu and Saka [47] and Mohan et al. [25] as optimum for the maximum sugar release within the LHW treatment of Japanese beech and bamboo, respectively.

### Comparison between TS and TP LHW hydrolysis

The overall mass balance after LHW hydrolysis was better conserved for cellulose as compared to hemicellulose and for raw TP and TS as compared to PT-TP and PT-TS, respectively (Tables 3, 4). This result was based on the sum of the yields of cellulose and hemicellulose between the liquid and solid fractions of the LHW hydrolysates, separately, from raw and pretreated tomato by-products.

Generally, LHW treatment is known to solubilize mainly the hemicellulose fraction of biomass [4]. Therefore, TS which contained much more hemicellulose tended to submit more hydrolysis as compared to TP. This fact was confirmed based on the total solubilization yields, expressed as weight losses in Tables 3 and 4. Effectively, LHW treatment at 200 °C resulted in the hydrolysis of 56.9 ± 0.8% of raw TS and 34.6 ± 1.6% of PT-TS, on dry basis, while only 31.5 ± 0.9% of raw TP and 22.9 ± 0.6% of PT-TP were solubilized at the same temperature. In the same line, the total lignin was much highly recovered in the solid residues of TP samples (raw TP and PT-TP) as compared to TS samples (raw TS and PT-TS). Hence, from this study it can be said that TP had a much more rigid lignocellulosic structure than TS, maybe because they have higher cellulose and lignin contents.

Although LHW treatment of PT-TP and PT-TS did not promote high yields of sugars within the liquid hydrolysates, as shown in Tables 3 and 4, LHW liquid fractions from raw TP and TS, treated specifically at 160 °C, seemed to be more promising for valorization as a source of bioethanol production [26, 49]. Thus, an extensive techno-economical study would be of high interest to determine the feasibility of utilizing these industrial by-products for ethanol production. Importantly, a compromise should be made between carotenoid, oil, and protein extractions and sugars hydrolysis. A supplementary enzymatic hydrolysis step might be efficient for improving the hydrolysis of sugars from cellulose and hemicellulose fractions of PT-TP and PT-TS [4, 37, 48]. Moreover, a flow-through method can be investigated for the hydrolysis of tomato industrial by-products, as this reactor configuration had been considered superior to batch methods for the solubilization of biomass in LHW treatment [27]. One other possible perspective could be the use of acid- or base-catalyzed LHW treatment of tomato by-products as suggested by Del Campo et al. [20]. The recovered lignin within the solid fraction after LHW treatment can be assessed as a food additive regarding its dietary fiber advantages [6]. The present analytical results suggest further investigation of the same cascade biorefinery processing of TP and TS together, avoiding the sieving step, and the integration feasibility of all thermal separation processes in a kind of “one stop unit.”

## Conclusions

This paper suggested an innovative biorefinery concept producing high-value compounds from tomato industrial residues. The work aimed to integrate SC-CO_2_ extraction of carotenoids and TSO, protein isolation from TP and TS, and LHW treatment of the residual biomass. Results indicated that tomato by-products are not only a green source of lycopene-rich oleoresin and TSO and of protein with good nutritional quality but also a potential feedstock for bioethanol production. As growing research has focused on LHW hydrolysis of lignocellulose for subsequent ethanol production, the present results would provide valuable practical experience regarding the hydrolysis of tomato industrial by-products.

## Abbreviations

TP: tomato peels
TS: tomato seeds
LHW: liquid hot water
SC-CO_2_: supercritical CO_2_
SFE: supercritical fluid extraction
PER: protein efficiency ratio
HMF: hydroxymethylfurfural
TSO: tomato seed oil
PT-TP: pretreated TP: tomato peels previously submitted to oleoresin extraction using SC-CO_2_ and protein batch extraction
PT-TS: pretreated TS: tomato seeds previously submitted to oil extraction using SC-CO_2_ and protein batch extraction
TP-120: TP submitted to LHW at 120 °C
PT-TP-120: PT-TP submitted to LHW at 120 °C
TS-120: TS submitted to LHW at 120 °C
PT-TS-120: PT-TS submitted to LHW at 120 °C
